# Nanopore Sequencing for HPV in Oropharyngeal Squamous Cell Carcinoma and Benign Tonsil Specimens

**DOI:** 10.1002/ohn.70240

**Published:** 2026-04-14

**Authors:** Mikayla G. Hubbard, Guang‐Sheng Lei, Robert Emerson, Danielle Schreiber, Thomas E. Davis, Diane W. Chen

**Affiliations:** ^1^ Department of Otolaryngology–Head and Neck Surgery Indiana University School of Medicine Indianapolis Indiana USA; ^2^ Department of Pathology and Laboratory Medicine Indiana University School of Medicine Indianapolis Indiana USA

**Keywords:** human papillomavirus, nanopore sequencing, oropharyngeal squamous cell carcinoma

## Abstract

**Objectives:**

Human papillomavirus (HPV) accounts for the majority of oropharyngeal squamous cell carcinoma (OPSCC) cases in the United States but understanding the prevalence of high‐risk HPV in oropharyngeal tissue remains poor. We evaluated nanopore sequencing, a novel rapid, and long‐read sequencing platform, on OPSCC tissue and tested it in benign tonsils from pediatric and young adult patients.

**Study Design:**

Cross‐sectional study.

**Setting:**

Academic tertiary referral center.

**Methods:**

Formalin‐fixed, paraffin‐embedded (FFPE) specimens of HPV‐positive and negative OPSCC diagnosed from 2013 to 2023 were sequenced on the nanopore MinION platform using an amplicon‐based approach. Nanopore sequencing was subsequently performed on FFPE tissue from benign tonsillectomy in patients ages 30 and younger performed from 2016 to 2023.

**Results:**

In 54 OPSCC cases, nanopore sequencing detected HPV DNA in all 27 HPV‐positive specimens with 96% of cases exhibiting high abundance (HPV reads >1000). HPV DNA was also detectable in all 27 HPV‐negative specimens, but 89% had a low abundance. By interpreting high HPV abundance as HPV‐positive, nanopore sequencing demonstrated a 96% sensitivity, 89% specificity, 90% positive predictive value, and 96% negative predictive value rate. In 150 benign tonsillectomy cases, 27% had HPV DNA detected by nanopore sequencing, all in low abundance, including 37% of patients younger than 10 years old. HPV DNA sequences detected belonged to subtype 16.

**Conclusions:**

Nanopore sequencing demonstrated high sensitivity and specificity for HPV DNA in FFPE OPSCC tissue. High‐risk HPV prevalence in pediatric and young adults may be higher than previously studied.

Human papillomavirus (HPV) is the leading cause of oropharyngeal squamous cell carcinoma (OPSCC) in the United States, now surpassing HPV‑associated cervical cancer in incidence.^1^, ^2^, ^3^ HPV‑mediated OPSCC is associated with significantly improved survival and treatment response compared with HPV‑negative disease, leading to its recognition as a distinct clinical entity and prompting ongoing investigation into treatment de‑intensification strategies aimed at reducing morbidity without compromising outcomes.^4^, ^5^, ^6^, ^7^, ^8^

HPV‑mediated oncogenesis through the E6 and E7 oncoproteins is well characterized; however, the temporal progression from viral exposure to tissue infection, integration, and malignant transformation remains poorly understood. Oral HPV prevalence is estimated at approximately 5% to 7% in adults based on oral swab studies, though such methods do not accurately reflect tissue‑level infection or cancer risk.^9^ Limited data exist regarding HPV prevalence within oropharyngeal tissue, particularly in pediatric populations, with prior studies reporting wide variability in tonsillar HPV detection.^10^ While HPV exposure is necessary for the development of HPV‑associated OPSCC, the likelihood that an infection will progress to oncogenesis remains unknown.^11^, ^12^

Current clinical HPV testing relies primarily on p16 immunohistochemistry as a surrogate marker secondary to the interaction of HPV oncoproteins in inactivating the tumor suppressor protein retinoblastoma (Rb), with DNA‑based assays reserved for confirmatory testing due to increased cost and turnaround time.^13^ Although p16 demonstrates high sensitivity, its specificity is limited, and combined DNA‑based methods offer improved diagnostic accuracy.^14^

Nanopore sequencing is an emerging third‐generation sequencing platform capable of rapid, cost‐efficient, long‐read DNA analysis (Oxford, UK). Its use has been implicated in the detection of point mutations, splicing variants, copy number alterations, structural variations, and epigenetic markers.^15^ While fresh tissue has been purported as optimal, recent studies demonstrate the feasibility of using formalin‐fixed paraffin‐embedded (FFPE) specimens.^16^, ^17^, ^18^ Hernandez et al recently validated high sensitivity and accuracy in nanopore sequencing for HPV using FFPE from presumed HPV‐mediated OPSCC specimens in addition to anogynecologic cytology specimens.^19^

Nanopore sequencing is in its infancy stage with its application in clinical oncology in HPV‐related cancers; its application with head and neck oncology needs further expansion and validation. Recent studies have utilized nanopore sequencing in elucidating viral DNA integration patterns into host DNA.^20^, ^21^ In this study, we sought to determine the feasibility of nanopore sequencing in the detection of HPV DNA in FFPE of HPV‐mediated and non‐HPV‐mediated OPSCC tissue and apply this platform to benign pediatric and young adult tonsil tissue to better characterize tissue‐level HPV prevalence.

## Methods

### Case Selection

This study was approved by the Institutional Review Board of Indiana University (IRB 19071) and the Eskenazi Health research review committee. Formalin‑fixed, paraffin‑embedded (FFPE) OPSCC specimens diagnosed between January 2013 and February 2023 were retrieved from the Indiana University Health pathology archives. HPV status was determined by prior p16 immunohistochemistry and/or high‑risk HPV in situ hybridization, and cases with insufficient DNA were excluded. Following the determination of nanopore sequencing feasibility, FFPE tissue from 150 tonsillectomies performed in patients aged 30 years or younger between 2016 and 2023 was analyzed. Multiple tissue blocks per patient were sequenced, with the highest HPV read count reported per individual.

#### DNA Extraction and Amplification from FFPE Tissues

In each tissue block, 3 sections of FFPE tissue, 10 µm thick each, were collected in 1.5 mL microcentrifuge tube. DNA was purified using QIAamp® DNA FFPE Advanced Kit (Qiagen GmbH, #56604) according to the manufacturer's instruction to achieve deparaffinization, Proteinase K lysis, decrosslinking, and RNA removal. The lysate was transferred to QIAamp MinElute column for DNA binding. DNA was eluted using 30 µL of H_2_O.

#### Multiplex Sequencing Library Preparation

**Figure 1:**
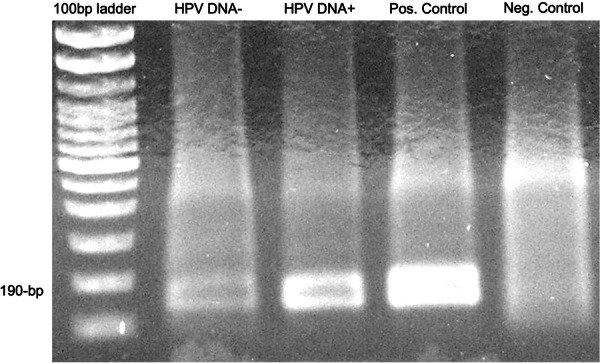
Gel electrophoresis detection of HPV DNA from amplicon‐based PCR. Lanes include the Trackit™ 100 base pair (bp) ladder, three HPV‐positive samples, and one HPV‐negative sample. 190‐bp indicates HPV (150‐bp target gene plus 40‐bp adapter sequence). HPV, human papillomavirus; PCR, polymerase chain reaction.

To prepare DNA library for sequencing, A two‐step PCR approach, tailed amplicon PCR and barcoding PCR, were carried out (Figure 1). The first‐round tailed amplicon PCR was performed using the extracted DNA as a template. A 150‐bp DNA in L1 region of HPV was amplified with tailed primers, GP5+/6+ Forward‐tailed 5′‐TTTGTTACTGTGGTAGATACTACTGTAGCTGTACCTGGTTCCT‐3′, and GP5+/6+ Reverse‐tailed 5′‐ GAAAATAAACTGTAAATCATATTCCAGCGAAATGTGTCGAAGAAAG‐3′ (the underlined sequences are HPV locus‐specific, the extra sequences are for the universal primers of barcoding PCR), and Terra^TM^ Direct PCR Red Dye Premix (#639286; TaKaRa Bio. The thermocycler temperature and time were set as follows: initial denaturation at 98°C for 2 minutes, followed by 50 cycles of 98°C for 10 seconds, 60°C for 15 seconds, and 68°C for 30 seconds. The second‐round barcoding PCR was performed using the tailed amplicon DNA as template and the PCR Barcoding Expansion kit (EXP‐PBC096; Oxford Nanopore Technologies) to incorporate a unique barcode into each tailed amplicon. The barcoding PCR amplification was following the cycling conditions: initial denaturation at 98°C for 2 minutes, followed by 15 cycles of 98°C for 10 seconds, 60°C for 15 seconds, and 68°C for 30 seconds. The barcoded amplicons were pooled together then extracted using QIAquick PCR Purification Kit (#28104; QIAGEN).

#### Ligation Sequencing, Base Calling, Data Analysis

The barcoded DNA libraries (100 fmol) were performed end‐prep using NEBNext® Ultra II End Prep Enzyme Mix (E7546, NEB) then following the adapter ligation using Oxford nanopore Ligation Sequencing Kit V14 (SQK‐LSK114; Oxford nanopore Technologies). Multiplex sequencing was performed using a Flongle R10.4.1 flow cell (FLO‐FLG 114, ONT) with a MinION (ONT) device according to the manufacturer's protocol. The base‐calling was carried out using MinKNOW software. The data were uploaded to the cloud‐based analysis platform EPI2ME (ONT). The metagenomics workflow in EPI2ME was selected for real‐time analysis. Sequencing results from EPI2ME were cross‐referenced with nucleotide BLAST for HPV subtyping.^22^ Total run time from DNA extraction was approximately 12 hours.

#### Comparative Assay With Quantitative PCR

A third assay using quantitative PCR (qPCR) for HPV DNA detection was performed for internal validation. Two additional ribbons of FFPE from each sample were collected. DNA was purified using Maxwell DNA FFPE kit (#AS1450; Promega) according to the manufacturer's instruction. DNA purified from head and neck FFPE tissue was then amplified in a multiplex qPCR reaction to detect HPV types 16 and 18 using the same primers. The quantity and quality of DNA was measured using the ProNex® DNA QC Assay on the Bio‐Rad CFX96 Touchor CFX Opus 96 according to the Technical Manual (TM513). A cycle quantification (Cq) below 30 was considered the threshold for a positive HPV‐mediated result.

## Results

### OPSCC cohort

Of the 58 archival OPSCC samples collected, 54 subjects had sufficient DNA for nanopore testing. There were 27 cases each of HPV‐positive and HPV‐negative OPSCC based on prior histological designation (Table 1). For HPV‐positive OPSCC cases, all specimens had HPV DNA detected by nanopore platform with a median 5688 reads (interquartile range [IQR] 4931‐6511). All HPV‐negative OSPCC cases had some HPV DNA detected (range 1‐4785 reads) with a median of 12 reads (IQR 7‐66).

**Table 1:** Nanopore Sequencing Results in HPV‐Positive and Negative Oropharyngeal Squamous Cell Carcinoma

Subject No.	HPV status	HPV reads	Total DNA reads	HPV:Total DNA reads ratio
1	Negative	4	91	0.04
2	Negative	12	97	0.12
3	Negative	13	271	0.05
4	Negative	52	991	0.05
5	Negative	14	102	0.14
6	Negative	1	229	0.00
7	Negative	7	887	0.01
8	Negative	3	62	0.05
10	Negative	7	266	0.03
11	Negative	20	123	0.16
12	Negative	697	1057	0.66
13	Negative	9	75	0.12
14	Negative	8	81	0.10
15	Negative	4	332	0.01
16	Negative	11	81	0.14
17	Negative	205	849	0.24
18	Positive	4618	6261	0.74
19	Negative	668	1287	0.52
20	Negative	29	103	0.28
21	Negative	4785	6854	0.70
22	Positive	7061	9819	0.72
23	Positive	5688	7951	0.72
24	Positive	5404	7733	0.70
25	Positive	8207	11,735	0.70
26	Positive	6310	8994	0.70
27	Positive	5746	8197	0.70
28	Positive	7330	10,016	0.73
30	Positive	7110	10,000	0.71
31	Positive	4830	6927	0.70
32	Negative	3,306	5254	0.63
33	Positive	6	465	0.01
34	Negative	9	91	0.10
35	Negative	7	79	0.09
37	Positive	4961	7517	0.66
38	Positive	6377	9518	0.67
39	Positive	5793	8951	0.65
40	Positive	6634	8919	0.74
41	Positive	6388	9471	0.65
44	Positive	5025	8126	0.62
45	Positive	5542	9018	0.62
46	Positive	4907	730	0.67
47	Positive	5118	9315	0.55
48	Positive	9591	11,420	0.84
49	Positive	5786	8751	0.66
50	Positive	7884	11,650	0.68
51	Positive	3879	6381	0.61
52	Positive	4643	6984	0.67
53	Positive	5307	8046	0.66
54	Positive	2788	6075	0.46
55	Negative	7	538	0.01
56	Negative	79	1032	0.07
57	Negative	4	781	0.01
58	Negative	2491	4256	0.59
59	Negative	22	563	0.04

**Figure 2:**
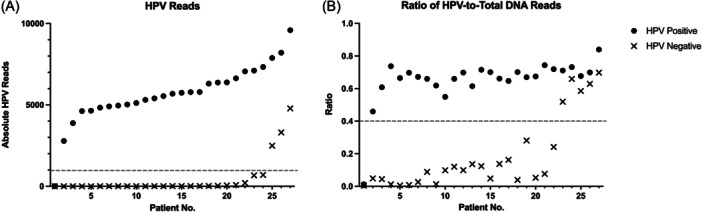
Nanopore results of HPV‐positive and negative OPSCC specimens. Scatterplot of (A) HPV reads and (B) HPV‐to‐total DNA ratio from nanopore sequencing in OPSCC cohort. The dashed line represents the threshold of high abundance. HPV, human papillomavirus; OPSCC, oropharyngeal squamous cell carcinoma.

Absolute and relative abundance of HPV DNA can be established by the number of HPV reads and the ratio of HPV‐to‐total DNA reads, respectively, meeting a threshold value. Guided by the pattern of results (Figure 2), abundance of HPV reads was interpreted as “high” for HPV reads >1000 and an HPV‐to‐total ratio >0.40. In the previously determined HPV‐positive samples by p16 or HPV ISH, high absolute abundance of HPV DNA was present in 26 of 27 (96%) cases (range 2788 to 9591 reads, median 5688 reads [IQR 4934‐6511 reads]) while one case (#33) had low abundance with only 6 HPV reads (Figure 2A). In the previously determined HPV‐negative cases, 24 (89%) cases had low absolute HPV abundance (range 1‐697, median 12 reads [IQR 7‐65.5 reads]) while three subjects (#21, 32, and 58) had a high absolute HPV abundance (range 2491‐4785).

When evaluating high relative abundance, 26 of 27 (96%) HPV‐positive cases had a ratio >0.40. In HPV‐negative specimens, 22 (81%) cases held a ratio <0.40 or low relative abundance (Figure 2B). Subject #19 demonstrated low absolute abundance (668 HPV reads) but high relative abundance (ratio 0.52).

**Figure 3:**
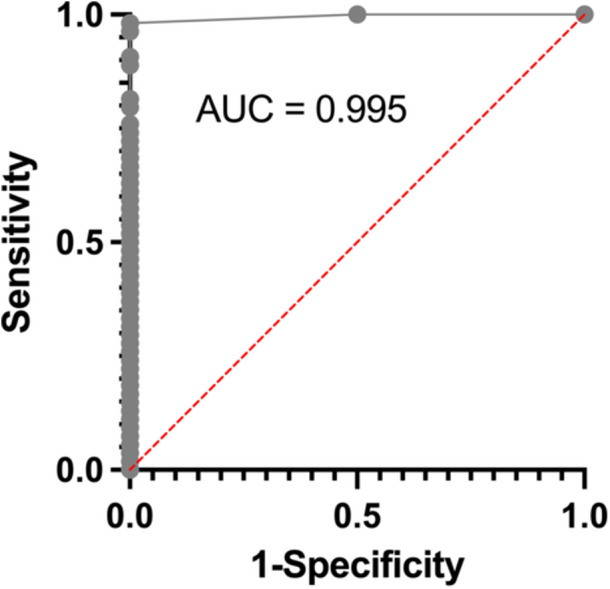
Sensitivity and specificity of nanopore sequencing utilizing absolute HPV DNA compared to p16 data. Area under the ROC curve is 0.995 (95% CI 0.986‐1.000, *P* < .0001). Red, dashed line indicates line of identity where AUC is 0.5. CI, confidence interval; HPV, human papillomavirus.

If defining HPV‐positive status is correlated to high absolute abundance of HPV reads, nanopore sequencing demonstrated 96% sensitivity, 89% specificity, 90% positive predictive value (PPV), and 96% negative predictive value (NPV) rate (Figure 3). If defining HPV‐positive status is paired with high relative abundance, nanopore sequencing demonstrated 96% sensitivity, 81% specificity, 84% PPV, and 96% NPV rate.

#### Comparison With qPCR

HPV status from qPCR correlated strongly with historical status and nanopore sequencing with a few deviations. In all HPV‐positive OPSCC specimens, HPV DNA was detected within the cycle quantification (Cq) threshold by qPCR. In the HPV‐negative OPSCC cohort, all but 6 patients had HPV DNA amplifiable by qPCR but at higher Cq than the accepted threshold of 30. For Subject #32, a non‐HPV‐mediated OPSCC, qPCR assay was positive for HPV (Cq 23), which correlated with the high abundance of HPV DNA detected by nanopore sequencing.

#### Benign Tonsillectomy Cohort

There were 150 cases (67% female, median age 19 [IQR 15‐25]) that yielded 313 FFPE blocks for nanopore testing. Nanopore sequencing detected HPV DNA in low abundance (range 1‐72 reads, median 17 [IQR 13‐29]) in 39 (26%) patients. Table 2 displays the nanopore sequencing results in patients with detectable HPV DNA. HPV DNA was detected in 10 (37%) of 27 patients younger than 10 years old (range 8‐55 HPV reads), 9 (18%) of 51 patients with ages 10 to 19 years old (range 2‐30 reads), and 20 (28%) of 72 patients with ages 20 to 30 years old (range 1‐72 reads). The relative abundance of HPV DNA was high in 3 patients under 10 years old (ratio range 0.48‐0.50) and in 5 patients in the second decade (ratio range 0.45‐0.50). HPV subtype 16 was detected in all HPV‐detectable samples.

**Table 2:** Nanopore Sequencing Detects HPV in 39 of 150 Benign Tonsil Specimens From Pediatric and Young Adult Populations

Subject No.	Age (Years)	Sex	HPV reads	Total reads DNA	HPV:Total reads ratio
10	6	M	13	27	0.48
11	24	F	9	80	0.11
12	6	M	21	57	0.37
13	28	F	1	8	0.13
24	7	F	15	2986	0.01
26	6	M	8	65	0.12
27	5	M	12	2856	0.00
33	29	F	24	7420	0.00
34	21	F	13	52	0.25
48	25	F	4	1499	0.00
54	25	F	28	85	0.33
56	28	F	57	113	0.50
59	20	F	7	22	0.32
61	26	F	45	94	0.48
62	30	M	13	41	0.32
65	8	F	49	76	0.65
66	7	F	27	58	0.47
67	30	M	20	41	0.49
73	18	M	2	43	0.05
76	21	F	43	115	0.37
77	26	F	20	91	0.22
79	19	F	14	106	0.13
83	23	F	44	113	0.39
89	18	F	25	95	0.26
111	18	F	30	92	0.33
132	29	F	54	116	0.47
134	25	F	8	24	0.33
137	18	F	5	30	0.17
138	16	M	17	73	0.23
139	14	M	14	47	0.30
143	23	M	23	51	0.45
145	7	M	55	111	0.50
146	23	F	29	91	0.32
147	6	F	29	80	0.36
148	24	M	11	44	0.25
149	15	M	14	73	0.19
150	17	M	4	588	0.01
151	27	F	11	1353	0.01

## Discussion

Nanopore sequencing is an emerging long‐read platform with potential clinical utility for HPV detection in OPSCC. Current HPV diagnostics rely primarily on p16 immunohistochemistry, with DNA‐based sequencing reserved for confirmatory testing due to cost and turnaround time. Prior nanopore studies in HPV‑related cancers have largely focused on tumors already classified as HPV‑positive or have relied on fresh tissue to characterize viral integration. Zhou et al performed genome‐wide analyses on fresh tissue to characterize virus‐human DNA integration events in HPV16‐positive cervical tumors.^20^ Similarly, Gauthier et al used fresh tumor tissue in eight patients diagnosed with HPV‐associated OPSCC by p16 immunohistochemistry to study HPV DNA integration events.^21^ In contrast, our study evaluates nanopore sequencing as a diagnostic tool and demonstrates its feasibility and validity for detecting HPV DNA in FFPE OPSCC specimens with high sensitivity and specificity. Additionally, this work expands prior findings by reporting HPV detection in nonviral‐mediated OPSCC, as well as applying this assay to benign tonsillectomy specimens. Our work also supports the validity of nanopore sequencing use on FFPE OPSCC as demonstrated by Hernandez et al while expanding on these findings by reporting HPV DNA reads on non‐HPV‐mediated OPSCC and by applying this assay to benign tonsillectomy specimens.^19^

The threshold at which low absolute HPV reads by nanopore sequencing reflect true tissue‑level infection versus artifact, or contamination remains unclear. Traditional quantitative PCR results detecting HPV DNA in all but 6 non‐HPV‐mediated OPSCC samples indicate high probability of true tissue‐level infection in the majority of the samples, The detection of HPV DNA by nanopore sequencing in HPV‑negative OPSCC specimens may have important implications. Low‑abundance HPV DNA is not inconsistent with a nonviral oncogenic process and supports the interpretation that tumor development was unlikely to be HPV‑mediated. These findings may also suggest that many individuals harbor low‑level latent HPV infection by mid‑to‑late adulthood, potentially due to cumulative oral exposure. Consistent with this possibility, approximately one quarter of pediatric and young adult patients in the benign tonsillectomy cohort demonstrated detectable HPV DNA within oropharyngeal tissue, which may represent early latent infection.^23^ Alternatively, low‑level HPV detection could result from contamination during tissue processing. Defining thresholds that distinguish true infection from background artifact will require further investigation.

Three patients (#21, 32, and 58) in the HPV‑negative OPSCC cohort demonstrated high absolute and relative HPV abundance by nanopore sequencing, raising concern for potential false‑positive results. One explanation for these findings may be the increased analytical sensitivity of nanopore sequencing compared with conventional diagnostic methods such as p16 immunohistochemistry, enabling detection of clinically occult HPV DNA. Alternatively, these tumors may harbor HPV DNA without HPV serving as the primary oncogenic driver. Additionally, two patients (#12 and 19) exhibited equivocal results, characterized by moderate HPV read counts in the 600 range with high relative abundance. These cases suggest the presence of a subset of HPV‑negative OPSCC patients with concurrent HPV infection and traditional carcinogenic risk factors, such as tobacco or alcohol exposure. In such instances, it remains unclear whether the detected HPV reflects latent infection or partial viral integration with limited oncogenic activity. Prior studies indicate that HPV‑positive OPSCC patients with significant tobacco exposure exhibit an intermediate‑risk phenotype with poorer outcomes, highlighting the complex interplay between viral and nonviral carcinogenic pathways.

For HPV‐positive OPSCC cases, subject #33 had only 6 HPV reads and a ratio of 0.01 while both p16 and HPV ISH were positive on pathology report. This may be possibly explained by the FFPE block retrieved having degraded DNA integrity, having too low DNA concentration for PCR amplification, or having the sample taken from a nonrepresentative portion of the tumor. DNA quantity and quality can degrade over time after the FFPE preservation process.^24^, ^25^ Mimosa et al suggested post‐PCR DNA concentration cutoff to be at least 5 ng/uL for nanopore sequencing.^17^ Despite having sufficient DNA postextraction, it is possible that this specimen had high fragmentation of DNA to accurately support an amplicon‐based approach.

There is temporal latency from initial HPV exposure to infection, integration, and oncogenic activation that remains poorly understood, particularly in OPSCC. Oral HPV prevalence is estimated at approximately 5% globally and in North America based on oral swab studies, though this does not correlate with the risk of developing OPSCC.^26^ In this study, detection of high‐risk HPV DNA in benign tonsillectomy specimens from very young patients represents a novel finding. Analysis of FFPE suggests that HPV infection within oropharyngeal tissue may be more prevalent than previously reported, with HPV DNA detected in 26% overall, including 37% of patients <10 years of age. Previous literature reports wide variability in pediatric tonsillar HPV prevalence, ranging from 0% to 21% and influenced by geographic location.^10^ Notably, the largest US‐based study prospectively analyzing oral swabs and fresh tonsil tissue found a 0% HPV infection rate, while earlier, PCR‐based studies reported low prevalence limited to low‐risk subtypes.^27^, ^28^ Collectively, our findings suggest the prevalence and timing of high‐risk HPV infection may be significantly underestimated.

The national completion rate of the HPV vaccination series among eligible adolescents is estimated to be 57%.^29^ Current guidelines recommend routine HPV vaccination at ages 11 to 12 years but may begin as early as 9 years of age.^30^ Routine vaccination is recommended through age 26, after which vaccination may be administered based on shared clinical decision‑making. In the benign tonsillectomy cohort examined in this study, 18% of patients were younger than 9 years and therefore below the recommended vaccination age, 63% were between 9 and 26 years and vaccine‑eligible, and 19% were older than 26 years. Although these findings may have important public health implications, particularly regarding the timing of HPV vaccination, further prospective studies are needed to validate nanopore sequencing performance and determine true HPV prevalence in pediatric populations. Future work should also aim to establish standardized thresholds distinguishing low‑level HPV DNA detection from true infection while accounting for potential artifact or contamination.

Nanopore sequencing demonstrates potential cost‑effectiveness when multiple samples are batched for simultaneous processing. Using a 96‑well array, the estimated cost per specimen was approximately $14 USD for the OPSCC cohort and $13 USD for benign tonsil tissue (Table 3). Chan et al reported using a 24‐well array with the cost of $51 USD per patient.^31^ Comparatively, HPV determination by p16 immunohistochemistry at our institution costs approximately $36.50 USD per specimen. Although nanopore sequencing enables high sample throughput and faster sequencing than traditional next‑generation sequencing platforms, the overall turnaround time from DNA extraction to data interpretation remains suboptimal for intraoperative applications such as surgical margin assessment. Further evaluation using fresh tissue is needed, particularly given the loss of tissue architecture during nucleic acid extraction. Notably, the HPV primers used in this study have also been successfully applied to anal and gynecologic FFPE specimens, suggesting broader multisite applicability.^19^

**Table 3:** Internal Cost Comparison of Nanopore Sequencing FFPE OPSCC and Benign Tonsils

OPSCC cohort		
Procedure	Number of specimen	Cost
DNA extraction and PCR	58 specimen blocks	$11.50 + USD × 58 specimen = $667 USD
Nanopore sequencing	58/96‐well = 1 run total	$121 USD/run × 1 run = $121 USD
	N = 96 (96 wells × 1 run)	
Cost per specimen		($667 + $121 USD)/58 specimen = **$13.59 USD** per specimen

Benign cohort		
Procedure	Number of specimen	Cost
DNA extraction and PCR	313 specimen (150 patients, varying no. blocks/patient)	$11.50 USD × 313 specimen = $3,600 USD
Nanopore sequencing	313 specimen/96 = 4 runs	$121 USD/run × 4 runs = $484 USD
	N = 384 (96 wells x 4 runs)	
Cost per specimen		($3600 + $484 USD)/313 specimen = **$13.05 USD** per specimen

### Limitations

This study has several limitations. The relatively small sample size limits generalizability and statistical power. DNA degradation in FFPE tissue may confound amplicon‑based sequencing and lead to underestimation of HPV abundance. Additionally, because nanopore sequencing is a relatively novel platform, no standardized threshold exists for defining HPV positivity. As a result, HPV abundance was used as an interpretive metric, with cutoffs for high abundance determined post hoc based on observed data patterns.

The study was not designed to determine whether absolute HPV read counts or relative abundance more accurately reflect true HPV status; therefore, both measures were reported, as each is commonly used in microbial DNA analyses. Additionally, distinguishing true HPV DNA presence from artifact or contamination remains challenging, particularly at very low read counts. This limitation may have contributed to overestimation of HPV prevalence in the benign tonsillectomy cohort. However, most patients (111 of 150) had no detectable HPV DNA, and positive cases demonstrated modest relative abundance and sufficient total DNA reads to support sequencing validity.

The study also lacked sufficient power to establish thresholds for differentiating contamination, processing error, or background noise. Use of de‑identified FFPE specimens further limited access to relevant clinical data, including vaccination status and history of HPV‑associated disease, like respiratory papillomatosis or other HPV‐driven cancer. Future studies can address these limitations and explore other applications such as circulating tumor DNA, though our OPSCC cohort preceded the time for which ctDNA could be sampled routinely.

## Conclusion

Nanopore sequencing demonstrated high sensitivity and specificity for HPV DNA using FFPE OPSCC tissue, but the diagnostic role of this platform requires further investigation. High‐risk HPV prevalence in pediatric and young adults may be higher than previously studied, warranting further studies in epidemiological and public health domains.

## Author Contributions

**Mikayla G. Hubbard**, study design, data analysis, manuscript drafting, final approval; **Guang‐Sheng Lei**, study design, data acquisition and interpretation, manuscript drafting and review, final approval; **Robert Emerson**, study design, data acquisition and interpretation, manuscript review, final approval; **Danielle Schreiber**, study design, data acquisition and interpretation, manuscript review, final approval; **Thomas E. Davis**, study design, manuscript review, final approval; **Diane W. Chen**, sudy design, data analysis and interpretation, manuscript drafting and review, final approval.

## Disclosures

### Competing interests

None.

### Funding source

None.
